# Ground glass opacity resection extent assessment trial **(GREAT)**: A study protocol of multi-institutional, prospective, open-label, randomized phase III trial of minimally invasive segmentectomy versus lobectomy for ground glass opacity (GGO)-containing early-stage invasive lung adenocarcinoma

**DOI:** 10.3389/fonc.2023.1052796

**Published:** 2023-01-19

**Authors:** Hanyue Li, Yiyang Wang, Yang Chen, Chenxi Zhong, Wentao Fang

**Affiliations:** Department of Thoracic Surgery, Shanghai Chest Hospital, Shanghai Jiao Tong University School of Medicine, Shanghai, China

**Keywords:** segmentectomy, lobectomy, ground glass opacity, early-stage invasive lung adenocarcinoma, minimally invasive surgery

## Abstract

**Background:**

With widely use of computed tomography (CT) screening, an increasing number of early-stage lung cancers appearing as ground glass opacity (GGO) have been detected. Therefore, attempts have been made to investigate the feasibility of segmentectomy instead of lobectomy for those patients with GGO. However, the two recently released phase III trials failed to distinguish between GGO-containing lesions from pure solid nodules in the inclusion criteria, and the surgical methods did not distinguish between minimally invasive surgery and open thoracotomy. In addition, total lesion size≤ 2cm was taken as the inclusion criterion, instead of the solid part size recommended in the eighth edition of Union for International Cancer Control/International Association for the Study of Lung Cancer/American Joint Committee on Cancer (UICC/IASLC/AJCC) staging system. Hence, this present trial aims to figure out whether minimally invasive segmentectomy shows superiority in perioperative outcomes and non-inferiority in oncological prognosis over minimally invasive lobectomy among patients with GGO-containing clinical stage T1a-T1b lung invasive adenocarcinoma (IADC).

**Methods/design:**

Sample sizes are 1024 patients, who will be randomized into minimally invasive segmentectomy and lobectomy groups . Patients will be collected from 19 hospitals in China. Patients with peripheral mixed ground glass opacity (mGGO) with 0.5cm<total lesion size ≤ 3cm and 0.5cm<solid component size ≤ 2cm in lung window on CT scan are enrolled. The primary endpoint is 5-year recurrence-free survival (RFS). The secondary endpoints are 5-year overall survival (OS), perioperative outcomes and pulmonary function preservation. Kaplan-Meier curves are plotted to compare the survival outcomes between the two arms. Subgroup analyses are also performed to investigate the benefit of segmentectomy among different clinical variables.

**Discussions:**

If the primary endpoint shows at least non-inferiority in 5-year RFS of segmentectomy to lobectomy, minimally invasive segmentectomy can be recommended as an alternative to minimally invasive lobectomy. If second endpoints show non-inferior 5-year OS along with better perioperative outcomes and/or better pulmonary function preservation of segmentectomy compared with lobectomy after the primary endpoint has reached, minimally invasive segmentectomy may become a preferred procedure for patients with GGO-containing clinical stage T1a-T1b IADCs.

**Trial registration:**

Chinese Clinical Trial Registry. Trial registration number: ChiCTR2000037065.

**Clinical trial registration:**

https://www.chictr.org.cn/, identifier ChiCTR2000037065.

## Introduction

Lobectomy has been recommended as the standard procedure for clinical stage I lung cancer less than 3cm since the Lung Cancer Study Group (LCSG) trial was published in 1995. Sublobar resections including anatomical segmentectomy were reserved as a palliative procedure for high-risk patients who were functionally unfit for standard lobectomy (1). Nowadays, the scenarios of both thoracic surgery and disease profile have changed dramatically. The results of the LCSG trial performed nearly three decades ago may not be suitable to guide current practice.

First, the LCSG trial did not separate anatomical segmentectomy from wedge resection when comparing sublobar resections with lobectomy. In a propensity score matching analysis, anatomical segmentectomy showed 20-30% survival benefit than wedge resection in patients with stage I non-small cell lung cancer (NSCLC) (2), indicating that the oncological efficacy of anatomical segmentectomy may be different from wedge resection.

Second, the disease profile of lung cancers is quite different from 30 years ago. With the widespread use of computed tomography (CT) screening (3), small-sized pulmonary nodules, especially those containing ground glass opacity (GGO) component, are increasingly detected. Different from pure solid pulmonary nodules, the GGO component of a subsolid nodule is corresponding to non-invasive histology, while the solid component is considered as the invasive part of the lesion (4). In consequence, anatomical segmentectomy is reconsidered as an intentional procedure for selected patients with peripheral nodule ≤ 2cm, including adenocarcinoma *in situ* or nodule≥50% GGO appearance on CT scan or with a long doubling time under radiologic surveillance (≥400 days) according to the The National Comprehensive Cancer Network (NCCN) guidelines (5). Accordingly, current indications of segmentectomy as an intentional procedure cover only carcinoma *in situ* (Tis), microinvasive adenocarcinoma (T1mi) and some of T1a lesions (GGO-containing lesions sized ≤2cm with GGO component >50% and solid component <1cm). Our previous study has found that segmentectomy showed similar oncological results to lobectomy for both T1a and T1b GGO-containing lung adenocarcinomas, as long as the total lesion size was ≤3cm and the solid component size was ≤2cm (6). Thus, further randomized controlled study is warranted to see whether segmentectomy shows oncological non-inferiority to lobectomy in patients with GGO-containing clinical stage T1a and T1b invasive lung adenocarcinoma.

Third, thoracic surgery has come into the era of minimally invasive surgery (MIS) including video-assisted thoracic surgery (VATS) and robotic-assisted thoracic surgery (RATS). Compared with open lobectomy, VATS lobectomy demonstrates a better recovery of physical function according to the VIOLET study (7). Besides, MIS presents less postoperative pain and better quality of life after surgery, due to the rapid development of surgical skills and minimally invasive technologies (8, 9). More importantly, long-term oncological outcomes of MIS procedure are at least not inferior to open thoracotomy for early-stage lung cancers (10, 11). Now, MIS is the preferred approach according to the guidelines.

For now, new evidences are in need to see if minimally invasive segmentectomy is at least oncologically non-inferior to lobectomy in patients with clinical stage T1a and T1b GGO-containing IADCs. Also, it is important to answer whether MIS segmentectomy is indeed superior in improving perioperative outcomes and pulmonary function preservation. For these reasons, we are performing a multi-institutional, prospective, open-label, randomized phase III trial comparing the results of minimally invasive segmentectomy versus lobectomy in patients with GGO-containing clinical stage T1a-T1b invasive lung adenocarcinoma. The main purpose of this article is to introduce the study protocol of this clinical trial.

## Patients and methods

### Study setting and objectives

This study is a prospective, multi-institutional, open-label, randomized controlled phase III clinical trial in patients with clinical stage T1a-T1b lung adenocarcinoma with GGO component under the eighth edition tumor, node, metastasis (TNM) classification. The aims of the study are to investigate whether minimally invasive segmentectomy is non-inferior in oncological outcomes, but is superior in preserving pulmonary function and/or reducing perioperative risks, against minimally invasive lobectomy. This multi-institutional clinical trial is undertaken by Shanghai Chest Hospital, together with Zhongshan Hospital of Fudan University, Huashan Hospital of Fudan University, the First Affiliated Hospital of Zhejiang University School of Medicine, the Second Affiliated Hospital of Zhejiang University School of Medicine, Jiangsu Province People’s Hospital of Nanjing Medical University, Fujian Medical University Union Hospital, Fujian Medical University Cancer Hospital, Peking Union Medical College Hospital, Sixth Medical Center of Chinese People’s Liberation Army General Hospital, Sir Run Run Shaw Hospital, Liaoning Cancer Hospital & Institute, Union Hospital of Tongji Medical College, the Fourth Hospital of Hebei Medical University, the First Affiliated Hospital of Chongqing Medical University, the Affiliated Cancer Hospital of Zhengzhou University, the First Affiliated Hospital of Anhui Medical University, Nanjing Drum Tower Hospital, Beijing Chaoyang Hospital, Capital Medical University.

This study has been approved by the Institutional Review Board of Shanghai Chest Hospital (IRB approval date: January 19, 2021 and approve number: IS2112) and other participant hospitals. This clinical trial has been registered at Chinese Clinical Trial Registry (ChiCTR) (Registration number: **ChiCTR2000037065**).

### Inclusion criteria

Patients will be eligible if they fulfill all the following inclusion criteria. (1) Patient age is between 20 and 80 years old; (2) The location of the primary lesion should be in the peripheral 1/3 pulmonary field, where both lobectomy and segmentectomy with satisfactory proximal margin can be achieved; (3) Segmentectomy includes combined segmentectomy, but the extent of segmentectomy should be <1/2 of the corresponding lobe; (4) The primary lesion should appear as GGO containing on thin-sliced CT scan. Maximum size of the lesion should >0.5cm and ≤3cm, with the solid component size >0.5cm but ≤2cm on lung window (corresponding to cT1a-b); (5) Patients with ipsilateral multiple nodules can be included only if there are no more than 2 additional GGOs in a different lobe from the primary lesion, and must be peripherally located that can be removed by wedge resection. If the secondary lesion is a pure GGO, the size should be <1cm. If the secondary lesion is a mixed GGO, the solid component size should be <0.5cm. At most two wedge resections are allowed during the simultaneous operation for the primary lesion; (6) Patients with contralateral lesions can also be included if there are ≤3 GGO-containing lesions, with a solid component size smaller than that in the primary lesion; (7) All patients should be functionally fit for standard lobectomy and segmentectomy, with an American Society of Anesthesiologists (ASA) score of I-III and spirometry results of FEV1 ≥1.2L, FEV1% ≥60%, DLCO% ≥60%;(8) All patients are voluntary to participate in this clinical trial and written informed consents should be signed (**Table 1**).

**Table 1:** Patient inclusion and exclusion criteria.

Inclusion criteria
1. Age between 20 and 80 years old;
2. Lesion locates in the peripheral 1/3 pulmonary field, and both lobectomy and segmentectomy (satisfied margin) can be done;
3. Segmentectomy includes combined segmentectomy, but the extent of segmentectomy should <1/2 of the lobe;
4. The primary lesion should appear as GGO containing on thin-sliced CT scan. Maximum size of the lesion should >0.5cm and ≤3cm, with the solid component size >0.5cm but ≤2cm on lung window;
5. Patients with ipsilateral multiple nodules can be included only if there are no more than 2 additional GGOs in a different lobe from the primary lesion, and must be peripherally located that can be removed by wedge resection. If the secondary lesion is a pure GGO, the size should be <1cm. If the secondary lesion is a mixed GGO, the solid component size should be <0.5cm. At most two wedge resections are allowed during the simultaneous operation for the primary lesion;
6. Patients with contralateral lesions can also be included if there are ≤3 GGO-containing lesions, with a solid component size smaller than that in the primary lesion;
7. All patients should be functionally fit for standard lobectomy and segmentectomy, with ASA score of I-III and spirometry results of FEV1 ≥1.2L, FEV1% ≥60%, DLCO% ≥60%;
8. All patients are voluntary to participate in this clinical trial and written informed consents should be signed;
Exclusion criteria
1. The primary lesion is in the right middle lobe; 2. Lesions requiring resection of more than 1/2 of the corresponding lobe, such as a basal segmentectomy and left upper lobe tri-segmentectomy;
3. Previous diagnosed malignancy within 5 years;
4. Unstable angina pectoris or myocardial infarction history within 6 months;
5. Stroke within 6 months; 6. Previous history of ipsilateral thoracic surgery.
Patient withdrawal
1. Benign lesions or AAH, AIS, non-adenocarcinoma cancer confirmed by intraoperative frozen section or postoperative final pathology;
2. Hilar or mediastinal lymph node metastasis confirmed by intraoperative frozen section;
3. Change of resection extent after randomization;
4. Patient requires to withdrawal or terminate the study for personal reasons.

GGO, ground glass opacity; FEV1, the forced expiratory volume in one second; DLCO, diffusing capacity of the lung for carbon monoxide; ASA, American Society of Anesthesiologists; AAH, atypical adenomatous hyperplasia; AIS, adenocarcinoma in situ.

### Exclusion criteria

Patients will be excluded if any of the following exclusion criteria is met. (1) The primary lesion is in the right middle lobe; (2) Lesions requiring resection of more than 1/2 of the corresponding lobe, such as a basal segmentectomy and left upper lobe tri-segmentectomy, to guarantee resection margin; (3) Previous diagnosed malignancy within 5 years; (4) Unstable angina pectoris or myocardial infarction history within 6 months; (5) Stroke within 6 months; (6) Previous history of ipsilateral thoracic surgery (**Table 1**).

### Patient withdrawal

Apart from inclusion and exclusion criteria mentioned above, those candidates will also be re-evaluated if any of the following exclusion criteria is appeared during the entire study. (1) Benign lesions or atypical adenomatous hyperplasia (AAH), adenocarcinoma *in situ* (AIS), non-adenocarcinoma cancer is confirmed by intraoperative frozen section or postoperative final pathology; (2) Hilar or mediastinal lymph node metastasis is confirmed by intraoperative frozen section; (3) Change of resection extent after randomization; (4) Patient requires to withdrawal or terminate the study for personal reasons (**Table 1**).

### Preoperative examinations

Once the candidate is qualified, the informed consent will be signed. The baseline characteristics of all the candidates, including family history, smoking history, comorbidity, symptoms and signs, would be collected. Before surgery, those qualified patients will take full preoperative examinations and evaluations, including chest CT, abdominal CT or ultrasound, cervical ultrasound examinations. Head magnetic resonance imaging (MRI) and positron emission tomography/computed tomography (PET.CT) are also preferred for the prevention of cancer metastasis, especially for patients with clinical T1b NSCLCs. Besides, preoperative pulmonary function examination as well as serum tumor markers will also be tested before surgery. In addition, each patient will receive simplified bedside pulmonary function examination the day before operation. All the preoperative examination and evaluation data will be entered into an investigator initiated trial Electronic Data Capture (IIT-EDC) System (Taimei Technology, Zhejiang Province, China) an electronic data collection system.

### Randomization

Central randomization will be performed and the patients will be randomized in a 1:1 ratio to either a minimally invasive anatomical segmentectomy group or a minimally invasive lobectomy group. Randomization will take place the day before surgery through a Central Randomization System based on the IIT-EDC.

### Surgery

Minimally invasive thoracic surgery including both VATS and RATS is required for this trial, including single-port, two-port, three-port or four-port incisions without rib spreading. Lobectomy and segmentectomy should be anatomically resected. The hilar structures should be separated and pulmonary vessels and bronchus should be divided respectively. Lobar fissure and intersegmental plane should be managed either by stapler alone or by stapler together with energy devices. Both segmentectomy and lobectomy groups should receive systemic lymph node dissection or sampling according to the NCCN guidelines (5).

**Figure 1:**
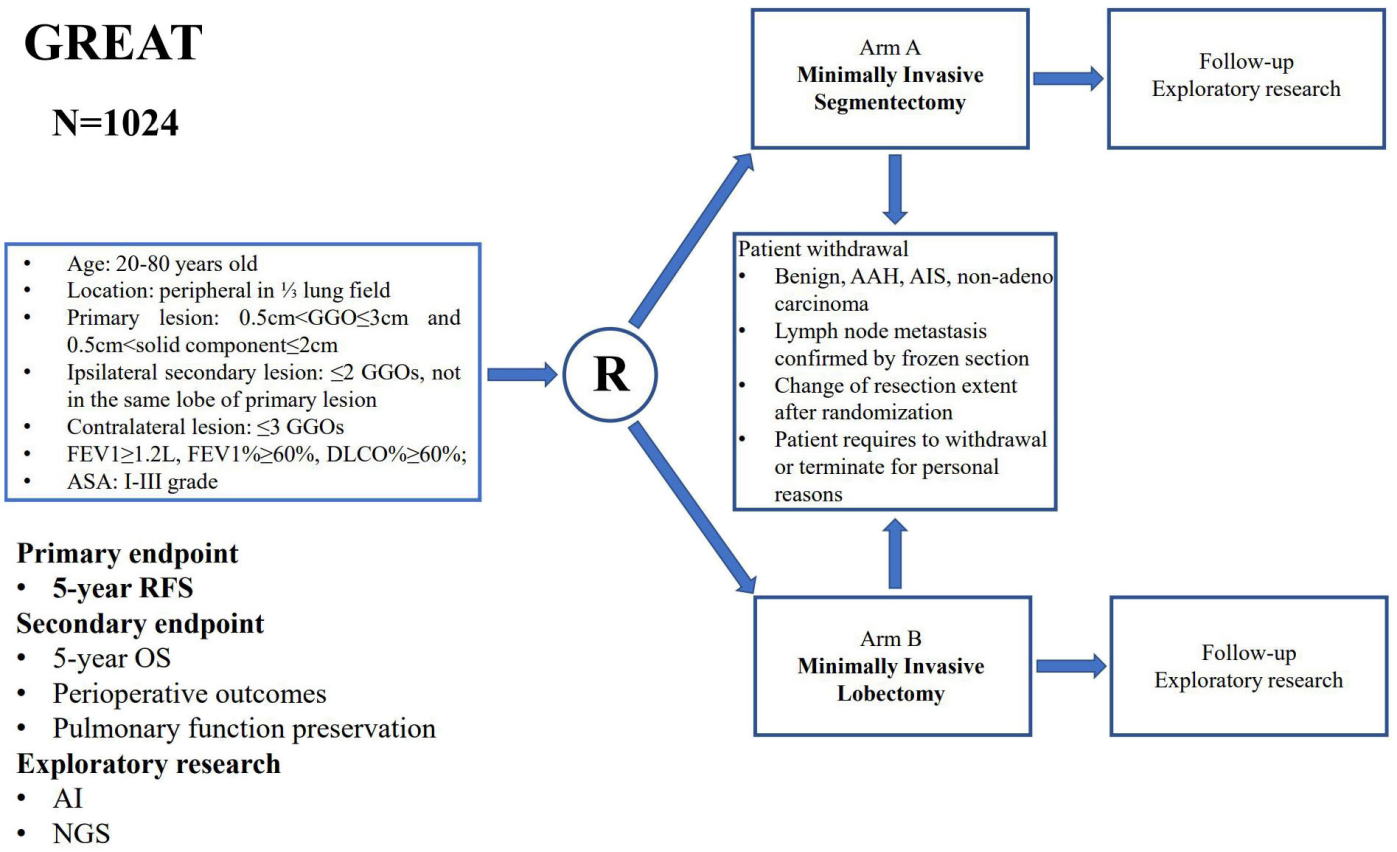
The study flowchart of the Ground Glass Opacity Resection Extent Assessment Trial (GREAT).

Intraoperative frozen pathology is required for the resected specimen, including the pulmonary surgical margin of segmentectomy. If the segmental resection margin is positive of malignancy on frozen section, segmentectomy should be converted to lobectomy. All the intraoperative data including intraoperative complications will be carefully recorded. In addition, preoperative imaging, peripheral blood sample and intraoperative tumor specimen of each patient will also be collected for further exploratory research (**Figure 1**).

### Postoperative managements

**Figure 2:**
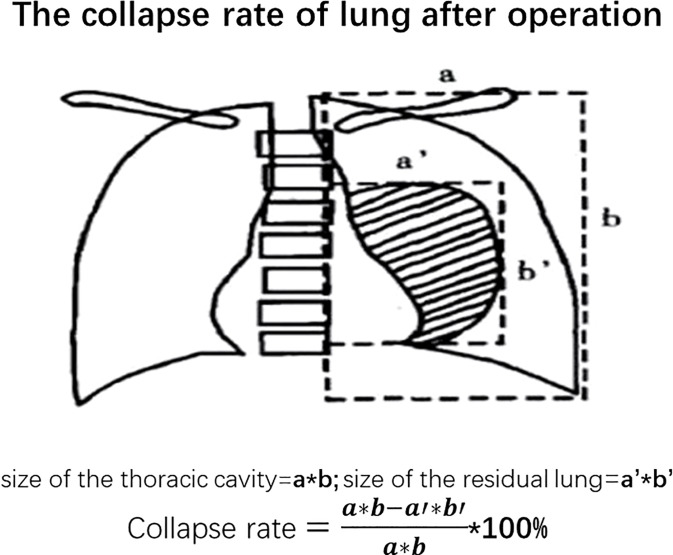
The collapse rate of lung after operation.

After surgery, digital drainage is preferred for chest drains to record the degree of airleak. Re-expansion of the residual lung will be calculated on chest X-ray on the first postoperative day (POD1) (**Figure 2**). Simplified bedside pulmonary function examination will be repeated on POD2. During the postoperative in-hospital interval, all the postoperative information along with complications will be recorded. The Common Terminology Criteria for Adverse Events (CTCAE) version 5.0 is used for determining postoperative complications where the severity ≥2 is considered as the occurrence of postoperative complications. Specifically, the definition of prolonged air leak is considered as air leak ≥5 days after pulmonary resection.

### Pathological evaluation

Pathological examinations include intraoperative fast frozen evaluation and postoperative final pathology examination. For frozen section, the resected specimen, the margin of resected bronchus and the pulmonary surgical margin of segmentectomy will be assessed during surgical operations by an experienced pathologist. Specially, for the pulmonary surgical margin of segmentectomy, the shortest distance between the tumor and the pulmonary surgical margin should be measured. In the final pathology, routine examinations should at least include number of tumors, tumor location, tumor size, pathological types and subtypes, status of pleural invasion (PI), status of lymphovascular invasion (LVI), status of spread through air spaces (STAS), numbers of resected lymph nodes and lymph node stations. Histological subtypes and subtype percentage of invasive adenocarcinoma should be all presented according to the World Health Organization (WHO) classification of lung tumors (12, 13). Gene mutations test by next-generation sequencing is encouraged for each participant.

### Postoperative adjuvant therapy

Whether postoperative adjuvant therapy is necessary will be evaluated by thoracic surgeons according to the final pathology of each patient. For patients with pathological stage IA NSCLC, routine follow-up strategy is recommended. For patients with pathological stage II-IIIA NSCLC and high-risk stage IB NSCLC (such as those with solid or micropapillary component, PI, LVI, STAS, etc.), platinum-based chemotherapy is recommended. Adjuvant targeted therapy and immunotherapy can also be considered for well-selected pathological stage IB-IIIA NSCLC patients.

### Follow-up

Each qualified patient after surgical resection should take postoperative follow-up in out-patient department. Within the first month after operation, patient will receive chest X-ray examination to assess the extent of residual lung expansion after radical resection. Pulmonary function examination *via* spirometry test will be performed in each participant at 6 months after surgery. In the first three years after surgery, patients need to receive chest CT scanning, neck and abdomen ultrasound examination and serum tumor markers every six months. Thereafter, the above examinations need to be repeated once a year until the fifth year after operation. All follow-up information is carefully recorded.

### Study endpoints

The primary endpoint is 5-year recurrence-free survival (RFS), calculated from the date of surgery to the date of cancer recurrence or patient death or the last follow-up. The secondary endpoints are 5-year overall survival (OS), perioperative outcomes and pulmonary function preservation. OS is calculated from the date of surgical resection to the date of patient death or the last follow-up. Perioperative outcomes include blood loss, operation time, morbidity rate, mortality rate, chest drain duration, length of hospital stay, and residual lung re-expansion. Pulmonary function change will be evaluated on short-term (the second day after surgical operation) and on long-term (6 month after operation) after surgery. 5% difference in FEV1 at 6 months after surgery is set as the secondary end point between the segmentectomy and the lobectomy groups. Exploratory research includes prediction of histological subtypes on preoperative imaging using artificial intelligence (AI). We also try to explore the characteristics of gene mutation status *via* next-generation sequencing (NGS) among GGO-containing early-stage NSCLCs.

### Sample size calculation

Expected 5-year RFS is 91% according to a previous study at Shanghai Chest Hospital (6). With a one-sided alpha error of 5% and at least 80% power and a non-inferiority margin of 5% along with a drop-out rate of 5%, the sample size of this study requires 1024 patients.

### Statistical analysis

A two-sided *p*<0.05 is set as a statistical difference. Pearson χ^2^ or Fisher’s exact test and Student t test are performed for the comparison of baseline characteristics, perioperative outcomes and the pulmonary functions. Comparisons of OS and RFS between minimally invasive segmentectomy and lobectomy groups are all estimated by Kaplan-Meier method and testified by the two-side Log-rank test. Cox proportional hazard regression model will be used to evaluate the hazard ratio of minimally invasive segmentectomy group and minimally invasive lobectomy group for OS and RFS, respectively. Subgroup analyses will also be performed by total lesion size ≤ 2cm or 2cm-≤3cm. Intention to treat analysis (ITT) will be performed for all enrolled patients. Per protocol analysis (PP) will be performed for patients who have completed the trial in compliance with the protocol.

## Discussion

In a systemic review and meta-analysis, intentional segmentectomy showed non-inferiority in overall survival and disease-free survival compared with lobectomy for patients with early-stage NSCLCs (14). Intentional anatomical segmentectomy has been transformed from palliative surgery into curative treatment for those well-selected patients. Nevertheless, whether anatomical segmentectomy can be accepted as the standard of care for patients with GGO-containing early-stage NSCLC is still controversial, mostly because of lack of evidences for its benefit on pulmonary function preservation and reduction in peri-operative risks in comparison with lobectomy.

Recently, two phase III trials comparing sublobar resections with lobectomy, including Cancer and Leukemia Group B (CALGB) 140503 and Japan Clinical Oncology Group (JCOG) 0802 have released follow-up results. First of all, in both CALGB 140503 and the JCOG 0802, lesions containing GGO were not distinguished from pure solid nodules (PSN) (15, 16). However, NSCLCs with GGO component demonstrate relatively indolent biological behavior than pure solid lesions including less lymph node involvement, rare distant metastasis and better oncological prognosis (6, 17). Therefore, mixing GGO-containing nodule and SPN together will affect the results of prognosis analysis. Our trial recruits only GGO-containing lesions. And the inclusion criteria of JCOG 0802 and CALGB 140503 were based on the 7th TNM staging system, which includes patients with nodule size ≤ 2cm and solid components ≤ 2cm (cT1a-bN0M0 according to 8^th^ TNM staging). This proposed trial, however, includes GGO-containing lesions with total diameter ≤ 3cm and solid components less than 2cm, which are also cT1a-bN0M0 but pertain to the 8^th^ edition TNM staging.

Apart from the differences in inclusion criteria, the surgery approach and resection extent are different in this designed trial. No surgical approach was specified in either CALGB 140503 and JCOG 0802, which means that minimally invasive and open surgery can all be used (15, 16). Thoracic surgery has come into the era of minimally invasive surgery, which helps to reduce surgical trauma and function loss compared with traditional open thoracotomy. So, in this trial, the surgical approach is limited to minimally invasive surgery. The extent of pulmonary resection has now become the dominant factor on surgical risks and functional recovery. Unlike the sublobar group of CALGB 140503, which did not distinguish between wedge resection and segmentectomy, patients in JCOG 0802 were only divided into segmentectomy group and lobectomy group. While in JCOG 0802, there was no restriction on number of segments resected in the segmentectomy group. Segmentectomy in the GREAT study must be less than 1/2 of the corresponding lobe. If the number of segments removed ≥1/2 of the total segments of corresponding lobe, the patients will be withdrawn from the study.

Theoretically, when less lung tissue is removed, more lung function may be retained. However, pulmonary function preservation has failed to reach the expected level of 10% in the JCOG 0802 trial (16). From our previous study, if the number of segments removed ≥1/2 of the total segments of corresponding lobe, then the lung function loss of segmentectomy is similar to that of lobectomy (18). And in this previous observational study, VATS segmentectomy could reserve about 4-5% more FEV1 than corresponding VATS lobectomy. So, in the GREAT trial, segmentectomy is expected to have 5% less FEV1 loss than lobectomy.

Only with such evidences can early-stage lung cancer patients truly benefit from the advent of modern surgical techniques.

## Conclusions

If the primary endpoint shows at least a non-inferiority in 5-year RFS after surgery, minimally invasive segmentectomy can be recommended as an alternative to minimally invasive lobectomy. If the second endpoints show non-inferiority in 5-year OS along with better perioperative outcomes and/or better pulmonary function preservation of segmentectomy compared with lobectomy after the primary endpoint has reached, minimally invasive segmentectomy may become a preferred procedure for patients with GGO-containing clinical stage T1a-T1b IADCs.

## Data availability statement

The original contributions presented in the study are included in the article/supplementary material. Further inquiries can be directed to the corresponding author.

## Ethics statement

This study has been approved by the Institutional Review Board of Shanghai Chest Hospital (IRB approval date: January 19, 2021 and approve number: IS2112) and other participant hospitals. This clinical trial has been registered at Chinese Clinical Trial Registry (ChiCTR) (Registration number: ChiCTR2000037065). The patients/participants provided their written informed consent to participate in this study.

## Author contributions

Concept Proposal: WF. Survey and Data Summary: HL and YW. Data Collection, analysis and statistics: YC and CZ. Research Regulatory: WF. Writing – Draft, proofreading and editing: HL and YW. All authors contributed to the article and approved the submitted version.
